# Fgf and Sdf-1 Pathways Interact during Zebrafish Fin Regeneration

**DOI:** 10.1371/journal.pone.0005824

**Published:** 2009-06-08

**Authors:** Mohamed Bouzaffour, Pascale Dufourcq, Virginie Lecaudey, Petra Haas, Sophie Vriz

**Affiliations:** 1 Université Paris Diderot, Paris, France; 2 Unité INSERM U770, Paris, France; 3 Université Paris XI, Le Kremlin-Bicêtre, France; 4 EMBL Heidelberg, Heidelberg, Germany; Ecole Normale Supérieure de Lyon, France

## Abstract

The chemokine stromal cell-derived factor-1 (SDF1) was originally identified as a pre-B cell stimulatory factor but has been recently implicated in several other key steps in differentiation and morphogenesis. In addition, SDF1 as well as FGF signalling pathways have recently been shown to be involved in the control of epimorphic regeneration. In this report, we address the question of a possible interaction between the two signalling pathways during adult fin regeneration in zebrafish. Using a combination of pharmaceutical and genetic tools, we show that during epimorphic regeneration, expression of *sdf1*, as well as of its cognate receptors, *cxcr4a*, *cxcr4b* and *cxcr7* are controlled by FGF signalling. We further show that, Sdf1a negatively regulates the expression of *fgf20a*. Together, these results lead us to propose that: 1) the function of Fgf in blastema formation is, at least in part, relayed by the chemokine Sdf1a, and that 2) Sdf1 exerts negative feedback on the Fgf pathway, which contributes to a transient expression of Fgf20a downstream genes at the beginning of regeneration. However this feedback control can be bypassed since the Sdf1 null mutants regenerate their fin, though slower. Very few mutants for the regeneration process were isolated so far, illustrating the difficulty in identifying genes that are indispensable for regeneration. This observation supports the idea that the regeneration process involves a delicate balance between multiple pathways.

## Introduction

Epimorphic regeneration requires the mobilization, as well as the migration and the proliferation of progenitor cells capable of restoring the missing part. This phenomenon presents an interesting opportunity for studying the coordination between proliferation and patterning, as both processes must be strictly regulated in time and space to ensure the restoration of the size and shape of the missing part. A model of choice for gaining insights into regeneration is provided by the zebrafish caudal fin [1]–[3]. The Fgf pathway, among many others, is involved in this process. The fibroblast growth factors (Fgfs) have been shown to be associated with many developmental processes including antero-posterior patterning [4], mesoderm induction [5], angiogenesis [6], axon extension [7] as well as appendage formation [8]. The deregulation of the Fgf pathway leads to severe pathologies including tumorigenesis and stem cell disorder as observed in the myeloproliferative syndrome (EMS) [9]. Fgfs function through a set of tyrosine kinase receptor (RTKs) known as the fibroblast growth factor receptors (FgfRs), for which four members have been identified in vertebrates [10]. Upon FGF binding, the receptors homodimerize and are autophosphorylated, leading to the activation of the kinase activity. This triggers a cascade of intracellular signals ending in the activation of target genes in the nucleus. Even though the signal transduction mechanisms by which FGFs function have been well characterized [11], identification of the targets genes is still limited.

The Fgf pathway is also an essential regulator of blastema formation after appendage amputation in amphibians as well as in fish. In Xenopus limbs, FGF-10 is sufficient to reactivate regeneration at later stages of development, when limbs have lost their regenerative capacity [12], while FGF-2 is able to stimulate *in ovo* chick limb regeneration [13] and to support regeneration of denervated axolotl limbs once blastema formation is initiated [14]. In zebrafish, a cocktail of Fgf ligands and FgfRs are induced during blastema formation and regenerating fin outgrowth [1], [2]. In particular, FgfR1 is expressed in pre-blastema mesenchymal cells during blastema formation, and maintained in subpopulations of blastemal and epidermal cells during outgrowth [15]. It has also been demonstrated that FgfR1 regulates blastemal cell proliferation during fin regeneration [15], [16]. Moreover, in an *fgf20a/dob* null mutant, no fin regeneration occurs as a consequence of an abnormal epithelialization and subsequent inhibition of blastema formation [17]. *fgf20a* expression is induced as soon as 6 hours after amputation (hpa) at the epithelial-mesenchymal boundary and in the blastemal cells, reaches the highest level at 24 hpa, and declines afterwards [17]. Fgfs have also been shown to be required in epidermal cells for a complete epimorphic regeneration of the heart [18]. The Fgf signalling pathway also instructs position-dependent growth rate during fin regeneration [19]. Lee *et al*. proposed that the reduction in the amount of Fgf signalling is essential to slow down and then stop regeneration. Therefore, while Fgf signalling is essential to initiate blastema formation and growth, the level of Fgf expression must also be precisely regulated in order to limit the regeneration process. Recent data provide evidence that Wnt signalling participates in the regulation of *fgf20a* expression [20] and that Fgf20a activates transcription factors, such as Lef1, downstream of the Wnt pathway [17] therefore underlying a reciprocal signalling in the initiation of regeneration between the Fgf and Wnt pathways.

We previously demonstrated that Sdf1, a small (11 kDa) secreted protein, plays a critical role in epidermal cell proliferation during fin regeneration. Indeed, increased SDF1 (either by over expression after electoporation of DNA or direct protein injection) inhibits epidermal cell proliferation and further regeneration [21]. We also showed that the expression of *sdf1a* and its two receptors (*cxcr4a* and *cxcr4b*) is precisely regulated during the process of regeneration and that a modification of *sdf1a* expression profile inhibits the progression of the regeneration [21]. While in neuronal and glial cells Fgf signalling seems to modulate CXCR4 and SDF1 expression [22], in bone marrow stromal cells FGF2 accelerates SDF-1 mRNA decay [23]. Recently, it has been proposed that Fgf signaling regulates *cxcr4b* and *cxcr7* expression during zebrafish lateral line development [24], [25]. Altogether, these observations show that different signalling pathways repeatedly interact for the proper development or function of various organs, therefore leading us to address the question of the relationship between the Sdf1 and Fgf pathways during the fin regeneration.

Here, we show that the Fgf pathway regulates the expression of the chemokine *sdf1a* as well as its receptors *cxcr4a*, *cxcr4b* and *cxcr7*, and that in turn Sdf1a negatively regulates the expression of *fgf20a* and FGF target genes. These findings reveal a new and fundamental role for Sdf1a in the process of fin regeneration, first as a possible mediator of the Fgf proliferative activity in the blastema formation, and then in the amplification of a negative feedback loop initiated by Wnt5b which subsequently restricts Fgf20a activity to allow proper regeneration.

## Results

### Expression of *sdf1a*, *cxcr4a*, *cxcr4b* and *cxcr7*, is controlled by the Fgf pathway

**Figure 1:**
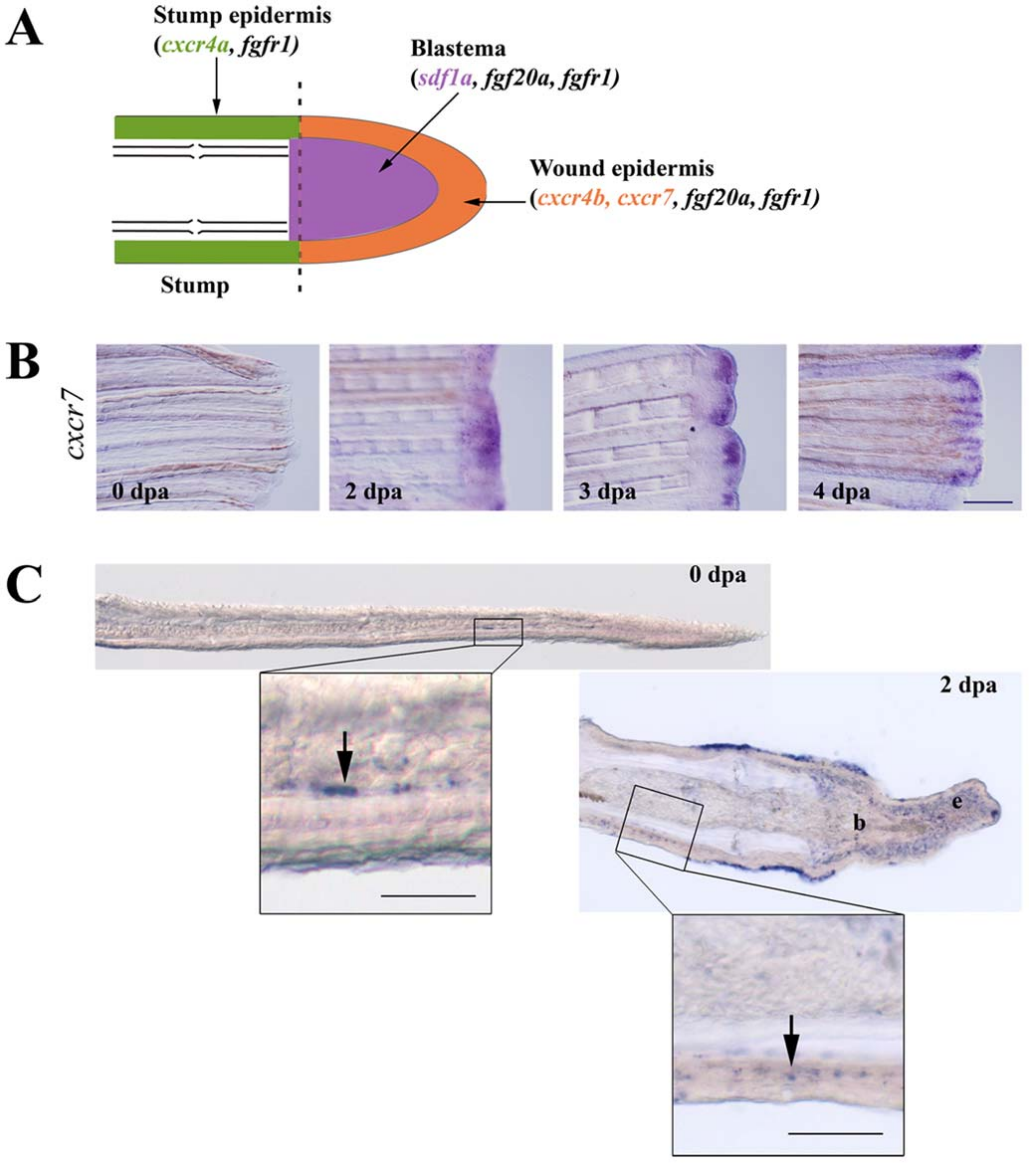
Expression of *sdf1* and its receptors during fin regeneration. A: schematic representation of FGF and SDF pathways in caudal fin regeneration. In green: stump epidermis expressing *cxcr4a* and *fgfr1*. In red: wound epidermis expressing *cxcr4b*, *cxcr7*, *fgf20a* and *fgfr1*. In blue: blastema expressing *sdf1a*, *fgf20a* and *fgfr1*. Longitudinal cross section through the dermal ray of a regenerating fin at 2 dpa. The dotted line indicates the amputation plane. B: *cxcr7* kinetics of expression during caudal fin regeneration. *cxcr7* mRNA expression pattern was analyzed by *in situ* hybridization on control fin (0 dpa) and in amputated fins allowed to regenerate for 2, 3 and 4 dpa. Scale bar, 100 µm. C: In situ hybridization for *cxcr7* on cryosections. *cxcr7* mRNA expression pattern was analyzed by *in situ* hybridization on cryosections of uncut fin (0 dpa) and in amputated fins allowed to regenerate for 2 dpa. Two days post amputation *cxcr7* mRNA is detected in the wound epidermis as well as in a few dispersed cells in the stump epidermis (enlarged view). Before amputation, only few mesenchymal cells show a staining for *cxcr7*. Scale bars, 50 µm.

**Figure 2:**
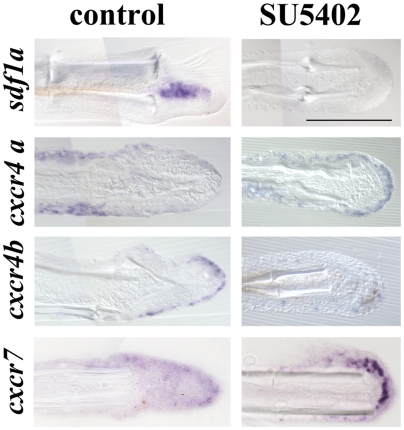
FgfR inhibition modifies *sdf1a*, *cxcr4a*, *cxcr4b* and *cxcr7* expression in ongoing fin regenerates. Sections of 2 dpa caudal fins from fish treated with DMSO (control) or FGFR inhibitor (SU5402) after *in situ* hybridization for *sdf1a*, *cxcr4a*, *cxcr4b* and *cxcr7*. Scale bar, 100 µm.

The zebrafish, as a consequence of a partial genome duplication, has two *sdf1* genes (*sdf1a* and *sdf1b*) [7] and two *cxcr4* genes (*cxcr4a* and *cxcr4b*) [26]. We have previously shown that only one of the ligands, *sdf1a*, is expressed in the regenerating fin while the two receptors (*cxcr4a* and *cxcr4b*) are expressed during the regeneration process (see[21] and Fig. 1A). More recently, another receptor of the same family, CXCR7/RDC1, has been shown to respond to SDF1 in human T lymphocytes [27]. In the zebrafish, this receptor is involved in two sdf1-dependent migration processes, lateral line formation and primordial germ cell location [28]–[30]. We therefore assessed the expression of *cxcr7* during fin regeneration by whole-mount *in situ* hybridization (ISH) (Fig. 1B) and by hybridization on cryo-sections (Fig. 1C). Whole mount *in situ* hybridization reveals that *cxcr7* is not expressed in adult fins, however amputation induces a transient and localized expression of this gene. The signal is first detected at 2 dpa in the wound epidermis and until 4 dpa in epithelial-mesenchymal boundary (Fig. 1B). Its expression then decreases and the *cxcr7* signal is not detectable after 5 dpa (data not shown). Hybridization on sections allows us to detect a few cells expressing *cxcr7* on the lateral border of the uncut fin and in a few mesenchymal cells of the stump (Fig. 1C). In order to investigate the possible regulation of Sdf1 and its receptors by the Fgf signalling pathway, activity of the FgfR was blocked using the drug SU5402, a drug first described as a specific inhibitor for FgfR1 phosphorylation [31]. It should be noted that SU5402 blocks FgfR1 activity by binding to a region well conserved in all four FgfRs, and so it might act on several of these receptors [32]. Caudal fins were amputated at the level of the first ray bifurcation and SU5402 was added to the water five hours after amputation. Fish were maintained in the drug during two days, and then the fins collected and expression of *sdf1a* and its receptors *cxcr4a*, *cxcr4b* and *cxcr7* analyzed by ISH (Fig. 2 and Fig. S2A). While *sdf1a* expression is normally detected in the blastema after amputation, it was undetectable after SU5402 treatment. Surprisingly, the expression of *cxcr4a*, which is usually induced in the epidermal cells of the stump shortly after amputation (see[21] and Fig. 2), was relocalized to the wound epidermis where *cxcr4b* is usually expressed after SU5402 treatment. In addition, *cxcr4b* expression, normally activated in the wound epidermis after amputation, failed to be induced when FgfR activity was blocked. Finally, *cxcr7* expression was strongly enhanced in the presence of SU5402 when FgfRs activity were blocked.

### SDF-1 signalling inhibits *fgf20a* expression

**Figure 3:**
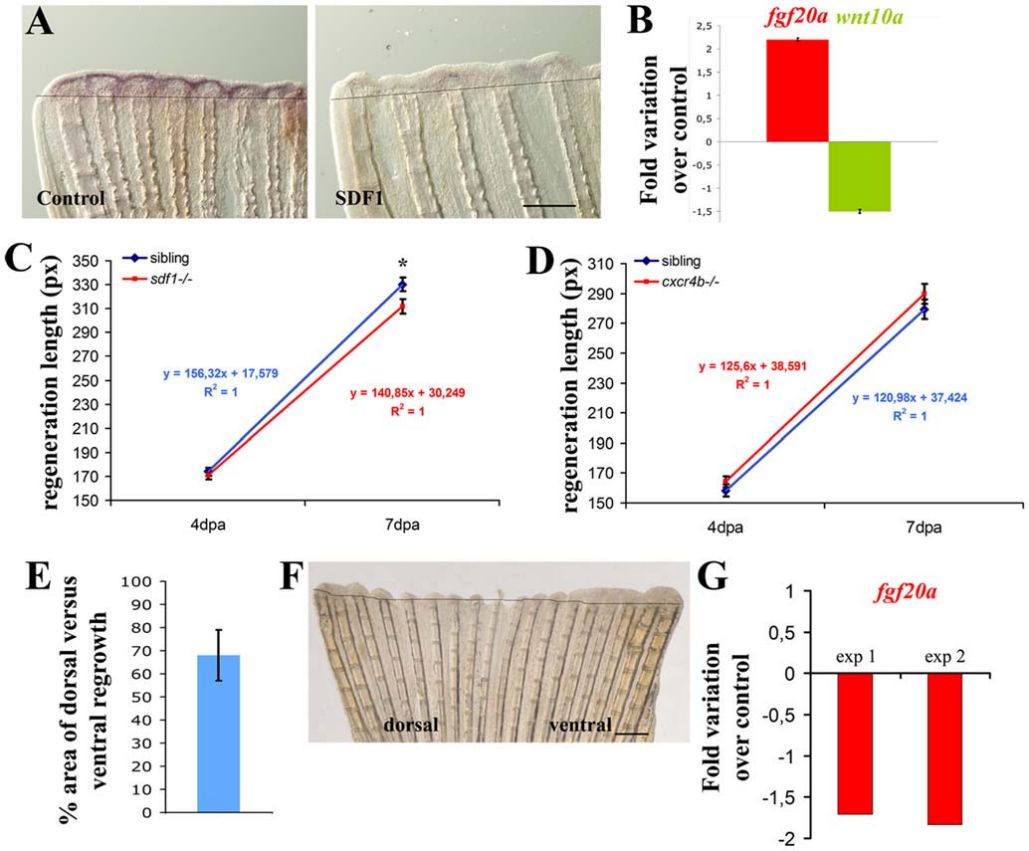
SDF1 inhibits *fgf20a* and activates Wnt10a expression during fin regeneration. A: Over-expression of SDF1 at the time of amputation turns off *fgf20a* expression. The protein SDF1, or BSA as a control, were injected into the fin at the time of amputation. Fins were allowed to regenerate for 48 hours before *fgf20a* expression was checked by ISH. Dotted lines demarcate amputation plane. Scale bar, 100 µm. B: *fgf20a* expression is enhanced in *medusa* mutant. *fgf20a* as well as *wnt10a* expression were analyzed by quantitative RT-PCR in *sdf1a/medusa* mutant and sibling fins at 48 hpa. *fgf20a* expression is increased 2.2±0.03 fold in *sdf1* mutant compared to siblings, while *wnt10a* expression is reduced 1.5±0.04 fold. Average values (±s.e.m.) from experiments performed in triplicate are presented. C, D: Fins regenerate slower in *sdf1/medusa* mutant compared to siblings (C) whereas *cxcr4b* mutant fish regenerates caudal fin with no abnormality (D). Length of the third dorsal and ventral regenerating fin ray of the regenerates were measured in *ody*−/− mutant fish and siblings (*ody*+/− and *ody*+/+) (D) and in *medusa/sdf1−/−* mutant fish and siblings (sdf1+/+ and sdf1+/−) (C) at 4 and 7 dpa. Precise measures were made in pixels (1 pixel corresponding to 3 µm). The average length of the regenerate allows to calculate the regeneration speed. No significant difference was observed between *ody*−/− mutant fish and siblings. Two independent experiments were performed. Experiment 1: n = 5 *ody−/−*, n = 7 siblings. Experiment 2 n = 5 *ody−/−*, n = 7 siblings (data not shown). Errors bars represent the s.e.m. of the average regenerate length. Fins regenerate slower in *sdf1/medusa* mutant n = 18 siblings (sdf1+/+ and sdf1+/−), n = 18 *medusa/sdf1−/−*. Errors bars represent the s.e.m. of the average regenerate length (* p<0.05). E–F: *cxcr7* overexpression inhibits blastema formation. Plasmid DNA expressing *cxcr7* (pCS2-cxcr7) was injected into the dorsal half fin whereas an empty plasmid (pCS2) was injected into the ventral part of the fin at the time of amputation prior to electroporation. Fins were allowed to regenerate for 48 hours before scoring blastema formation (n = 8). The percent area of dorsal versus ventral regrowth is presented in E and a representative fin in F. Scale bar, 500 µm. Dotted lines demarcate amputation plane. *G: cxcr7* overexpression inhibits *fgf20* expression. Plasmid DNA expressing *cxcr7* (pCS2-cxcr7) or an empty plasmid (pCS2) as control were electroporated into total fin at the time of amputation. Fins were allowed to regenerate for 48 hours before checking *fgf20* expression by quantitative PCR. Two independent experiments are presented in G. Experiment 1: n = 5; experiment 2: n = 5.

It has already been proposed that the Fgf pathway should be precisely regulated during fin regeneration [19], [20], so that the regeneration stops once the size of the former fin has been reached. In order to test a potential role for Sdf1 in the temporal control of the Fgf pathway, we sequentially used gain- and loss-of-function strategies. After injecting the SDF1 protein in the regenerating fin, *fgf20a* and FgfR1 target genes (*msxb*, *msxc* and *cxcr4a*, *cxcr4b* and *sdf1a*) expression was analyzed at 2 dpa. As expected from our previous study [21], caudal fins injected with the SDF1 protein show a regeneration profile that was strongly reduced compared to control fins (data not shown). Interestingly, this reduction was associated with a strong inhibition of *fgf20a* expression at 2 dpa (Fig. 3A). Expression of the Fgf target genes *msxb*, *msxc*, and *cxcr4b* was also strongly reduced at 2 dpa, while *cxcr4a* expression was localized to the margin of the fin in the wound epidermis (Fig. S3) as observed after treatment with the FGFR inhibitor (Fig. 2). These results strongly suggested that SDF1 negatively regulates *fgf20a* expression and as a consequence the FGF pathway during blastema formation. To confirm this result, we measured the expression level of *fgf20a* in the *medusa*/*sdf1a* mutant which are adult viable [28]. Quantitative RT-PCR performed in this background revealed a 2.2-fold upregulation of *fgf20a* expression at 2 dpa compared to siblings (Fig. 3B). Together these results show that SDF1 is capable of repressing *fgf20a* expression during the regeneration process. In addition, the level of expression of *fgf20a* was enhanced in SU5402 treated fins compared to DMSO treated fins (Fig. S2B). Since it is known that Wnt signalling is involved in the regulation of *fgf20a* expression during fin regeneration [20], we investigated the possibility that the expression of *wnt10a* was modulated by Sdf1. Quantitative RT-PCR analysis revealed a 1.5-fold reduction of *wnt10a* mRNA level in the *medusa*/*sdf1a* mutant background (Fig. 3B). This result suggests that repression of *fgf20* expression by SDF1 can be mediated, at least in part, by repression of Wnt10a expression. However the relatively small changes observed in gene expression also point to the complexity of the interaction network during blastema formation and growth.

### Respective roles of Cxcr receptors in the regeneration process

Partial fin amputation triggers the expression of all known SDF1 receptors, *cxcr4a*, *cxcr4b* and *cxcr7* ([21], Fig. 1). Since the expression domains of *cxcr4b* and *cxcr7* overlap with that of *fgf20a* [21] these two receptors were good candidates to mediate *fgf20a* inhibition. In addition, *cxcr7* is expressed later than *cxcr4b* after amputation making it the best candidate to downregulate *fgf20a*. To analyse further the respective roles of each of these receptors in the regeneration process, we quantified the regeneration rate in both *medusa/sdf1a^−^* and *ody/cxcr4b* mutant fish at 4 and 7 dpa and compared it to the rate siblings. In both cases the regeneration process occurs [21]. Whereas in *ody/cxcr4b* mutant fish, regeneration occurs at the same rate as in sibling fish (Fig. 3D), the size of regenerated fins in *medusa/sdf1a^−^* mutant fish was significantly shorter than in their siblings (Fig. 3C). This result supports a role for Cxcr7 rather that Cxcr4b in mediating the inhibition of *fgf20* expression. However, the absence of *Cxcr4b* function in *ody/cxcr4b* mutant might have been compensated via an ectopic upregulation of *cxcr4a*. This possibility is supported by our results showing that *cxcr4a* expression is indeed relocalized to the *cxcr4b* domain when Fgf signalling is blocked (Fig. 2). We thus directly tested this hypothesis by comparing the expression of *cxcr4a* in wild type and in *ody/cxcr4b* mutants. No difference in expression of *cxcr4a* was observed between *ody* mutant and wild type fish (Fig. S4). This indicates that *cxcr7* is the most likely effector in *fgf20* repression. Unfortunately, no *cxcr7* mutant is available to test directly this hypothesis. We thus decided to overexpress *cxcr7* in the fin from the beginning of regeneration. Fins were electroporated with a plasmid coding for *cxcr7* injected in one half fin or in the total fin at the time of amputation. Fins were then allowed to regenerate for 2 days before analysis. Cxcr7 overexpression induces a strong inhibition of blastema formation (Fig. 3E–F) as well as a reduction of *fgf20* expression (Fig. 3G).

## Discussion

Our results indicate that Fgf signalling, modulates the expression of *sdf1a* and its receptors during the regeneration process. First, we show that Fgf signalling is required in the blastema for the activation of *sdf1a* and *cxcr4b* expression and also in the stump epidermis for the activation of *cxcr4a*. Then we show that when Fgf signalling is blocked, both *cxcr4a* and *cxcr7* expression are ectopically activated in the wound epidermis. Interestingly, a comparison between *cxcr7* and *cxcr4b* expression profiles shows that both genes are expressed in the same domain but one after the other with *cxcr7* being detected when *cxcr4b* expression declines [21]. One hypothesis is therefore that cxcr4b is negatively regulating the expression of the two other G protein-coupled receptor cxcr4a and cxcr7. Interestingly, in the lateral line system cxcr4b and cxcr7 are also expressed in complementary domains [28], [29], and it has been proposed that cxcr4b represses the expression of cxcr7 in the leading region of the primordium [29].

It has been shown previously that the Fgf pathway and in particular FgfR1 is essential to the regeneration process [15]–[17] and that Sdf1, like FgfR1 is involved in epidermal cell proliferation during fin regeneration [21]. Our results give a new insight since they suggest that the chemokine Sdf1a could act as a mediator of Fgf signalling in order to promote epidermal cell proliferation [21], a process essential for the mechanism of regeneration.

Fgf signalling is required for initiating fin regeneration and controlling blastema formation [15], [17]. In addition, it has been recently shown that Fgf signalling instructs position-dependent growth rate during zebrafish fin regeneration [19]. This suggests that negative feedback progressively turns off Fgf signalling. Recent work demonstrates that Wnt10a promotes *fgf20a* expression in a β-catenin dependent manner, while Wnt5b down regulates *fgf20a* expression [20]. This elegant work shows that *wnt5b* is regulated by wnt/β-catenin signalling and provides the first example of negative feedback control of *fgf20a* expression during regeneration. The control of the Fgf pathway is very important for homeostasis in the organism. Even if the rate of regeneration is faster in *wnt5b/pipetail* mutant that in wild type fish [20], the difference suggests that another pathway is involved in the repression of *fgf20a* expression.

Using gain and loss of function approaches, we show here that SDF-1 signalling inhibits *fgf20a* expression. This pathway is therefore a good candidate to limit in time the action of FGF signalling pathway in parallel with Wnt5b. However, regeneration is only slightly inhibited in sdf1a−/− mutants. This discrepancy could be due to sdf1 signalling consecutively via two distinct receptors, Cxcr4b and Cxcr7. These two receptors have indeed been shown to respond very differently to the presence of SDF ligand in different biological contexts. For example, during both Primordial Germ cells (PGC) migration and lateral line formation in zebrafish, Cxcr7 has been proposed to act as a decoy receptor that sequesters sdf1 ligand making it unavailable for Cxcr4 [29], [30]. It is therefore likely that Cxcr4 and Cxcr7 have different and even opposite roles during the regeneration process. This would explain the subtle phenotype observed in sdf1 mutants. Altogether, this leads us to propose a model in which, upon amputation, *fgf20* expression is first activated, most likely by Wnt signals [20]; Fgf20 would then activate *sdf1a* as well as cxcr4b expression to promote the regeneration process. Downregulation of fgf20 expression would subsequantly allow cxcr7 activation. Finally we propose that SDF1a/Cxcr7 would exert a negative effect on *fgf20* expression to finely regulate the regeneration process.

**Figure 4:**
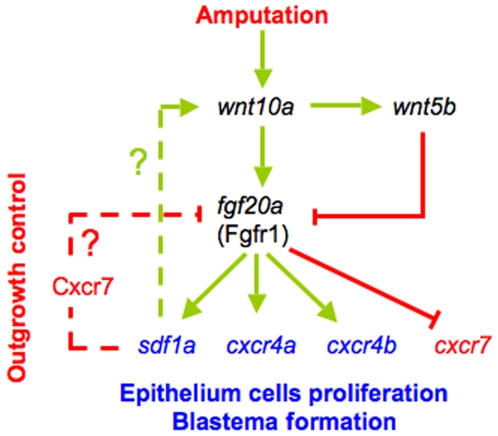
A model for signalling events regulating initiation of regeneration and fin outgrowth. Amputation of the caudal fin triggers *wnt10a* expression which then activates *fgf20* in a β-catenin-dependent mechanism [20]. Fgf signalling (most likely through FgfR1) activates the expression of the chemokine *sdf1* and its receptors *cxcr4a* and *b*, and then inhibits *cxcr7* expression. In return Sdf1a exerts a negative feedback on *fgf20a* expression in parallel to Wnt5b. We propose that as soon as the level of Fgf20a protein decreases, this allows *cxcr7* expression, Cxcr7 subsequently down regulating *fgf20a* in parallel to Wnt 5b. In this context the SDF1 pathway amplifies the negative regulation of *fgf20a* expression. In summary, Sdf1 thus plays two roles in fin regeneration. First it mediates Fgf activity to promote proliferation of epithelial cells [21], second it subsequently switches off Fgf activity by down regulating *fgf20* expression.

Very few mutants for the regeneration process were isolated so far, illustrating the difficulty to identify genes that have an indispensable function during regeneration. This observation supports the idea that the regeneration process involves a delicate balance between multiple pathways. Given the importance of shutting down the proliferation signal while regeneration is still ongoing, we expect significant redundancy between these pathways. We propose that Sdf1 plays a complementary role in this feedback mechanism (Fig. 4). In our model, the regeneration process is maintained as long as Fgf20a inhibits *cxcr7* expression. As soon as Wnt5b inhibits *fgf20a* transcription, the decrease of Fgf20a protein level, and therefore of Cxcr4b, would allow *cxcr7* expression. Cxcr7 can then be stimulated by Sdf1a to down regulate *fgf20a* in parallel with Wnt 5b. In this context the SDF1 pathway amplifies the negative regulation of the Wnt pathway on *fgf20a* expression. Finally, the decrease in Fgf20a levels induces in turn a decrease in Sdf1a level, thus closing the regulatory loop.

These findings underscore the complexity of the interaction network during blastema formation and growth. Although the SDF1/CXCR pathway and its functions were originally identified in the immune system, emerging data suggest that this pathway plays important roles in various functions like proliferation and migration of tissues during morphogenesis [33]–[38]. We propose a working model in which SDF1/CXCR cooperate with the Wnt pathway to control FGF signalling.

## Materials and Methods

### Fish care and surgery

Zebrafish colonies were maintained using standard methods [39]. The *odysseus* (ody) and the *medusa* mutants were previously described [28], [40]. For manipulation and amputation, adult zebrafish (5–10 months of age) were anesthetized in 0.1% tricaine (ethyl-m-aminobenzoate) and the caudal fins amputated using a scalpel. Regeneration was then allowed to proceed for various lengths of time. Fish were then anesthetized and regenerates were collected for further analysis.

### Quantitative RT-PCR

Total RNA was extracted from 5 caudal fins per experimental point using Trizol, according to the manufacturer's protocol (Invitrogen). 1 µg of total RNA was reverse transcribed by SuperScript II reverse transcriptase (Invitrogen) using oligo(dT) primers. Quantitative PCR was performed using ABI PRISM 7000 sequence detection system and SYBR green labelling system (Qiagen). Details concerning the parameters used are available on request. Gene expression level was normalized to *®-actin* or 18S rRNA. Each sample was tested in duplicate. Specific primers were designed by Primer 3 online software (http://frodo.wi.mit.edu/primer3/input.htm) to flank one intron in order to detect potential Genomic DNA contamination by melting curve analysis.

*®-actin* and 18S rRNA primer were previously described in [20]

*fgf20a*: forward (5′-TGTGGATAGCGGATTGTATCTG-3′), reverse (5′-ACCAATTCTCCTCAAACTGCTC-3′)

*wnt10a*: forward (5′-ATTCACTCCAGGATGAGACTTCATA-3′), reverse (5′-GTTTCTGTTGTGGGCTTTGATTAG-3′)

### Fin length measurements in *cxcr4b* and *sdf1a* mutant fish

*cxcr4b* (*odysseus, ody*) and *sdf1a* (*medusa*) homozygote and sibling regenerating fins were photographed at four and seven days post amputation. Then the dorsal and ventral third caudal fin ray was measured (from the amputation plane to the distal tip of the fin) using IMAGE J software (NIH) and the average length of the regenerate calculated for each fish.

### Fgfr inhibitor treatments

SU5402 (Calbiochem) was dissolved in DMSO and added to the fish water at a final concentration of 17 µM (0.01% DMSO) at 5 hpa. Tanks were maintained in the dark at 28°C until analysis.

### Misexpression of Sdf1 or Cxcr7

To overload Sdf1 ectopically in a regenerating fin, the proximal region of the caudal fin was either electroporated with a DNA plasmid expressing the zebrafish Sdf1a under the control of CMV promoter [16] or injected with purified human Sdf1 (Sigma) at 50 µg.mL^−1^, previously used on organ culture [41]. To overexpress Cxcr7, the caudal fin was electroporated with a plasmid DNA expressing the zebrafish cxcr7 under the control of CMV promoter (A detailed description of the cloning of the plasmid is available on request). For electroporation, the plasmid DNA (1 µg/µl) is injected into the dermal skeleton of anaesthetised adult zebrafish. Then ten 15 V pulses were administered with a 60 ms duration at 200 ms interval. Electric pulses were applied via a pair of electrode disks (7 mm diameter) rigged on the tips of tweezers (pinsettes-Type electrode 520, Quantum BTX instrument). Electrical contact with the fin skeleton was ensured by applying a conductive gel (aquasonic 100 ultrasound transmission gel). Square-wave electric pulses were generated by an ECM 830 BTX electroporator (Genetronics inc.). The manipulated fin was amputated 16 hours after DNA electroporation and subsequently collected for analysis. For evaluation of regenerative efficiency, we first measured the surface of the blastema and then normalized the values to the length squared of the amputation plane. For each experiment a set of ten fins was used.

### *In situ* hybridization and sections

DIG-labeled probes for *sdf1, cxcr4a*, *cxcr4b, cxcr7 and fgf20* were synthesized as previously described [21] and whole mount *in situ* hybridization was carried out using a robot (Intavis AG); details concerning the program used are available on request. For cross sections, fins were embedded in standard condition and section (20 µm) performed using a vibratome. *In situ* hybridization on cryo-sections was performed as described [42].

### Statistical analysis

Continuous variables are expressed as the mean±SEM. These variables were compared by using oneway analysis of variance and thereafter mean comparisons were made using Student t-tests adjusted to have an α level of 0.05. All statistical tests were two-tailed. *p* values that were less than 0.05 were considered to indicate statistical significance.

## Supporting Information

Figure S1 FgfR inhibition modifies, cxcr4a, expression in ongoing fin regenerates. Sections of 2 dpa caudal fins from fish treated with DMSO (control) or FGFR inhibitor (SU5402) after in situ hybridization for, cxcr4a. Scale bar, 100 µm.
(2.83 MB TIF)

Figure S2 A. SU5402 treatment modifies sdf1, cxcr4a, cxcr4b and cxcr7 expression. mRNA expression was analyzed by in situ hybridization on 2 dpa caudal fins from fish treated with DMSO (control) or FGFR inhibitor (SU5402). Scale bar, 100 µm. B. fgf20a expression is enhanced in SU5402 treated fins : fgf20a expression was analyzed at 48 hpa by quantitative RT-PCR in SU540-treated fish and DMSO fins as control. fgf20a expression is increased 1,9-fold when the FGF pathway is blocked compared to control. Average values (±s.e.m.) from experiments performed in quadruplicate are presented (** p<0.01).
(3.74 MB TIF)

Figure S3 SDF1 overexpression inhibits Fgf downstream genes expression The SDF1 protein, or BSA as a control, were injected in the fin at the time of amputation. Fins were allowed to regenerate for 48 hours before being stained for Fgf20 downstream genes cxcr4a, cxcr4b, msxb and msxc expression. Dotted lines demarcate amputation plane. Scale bar, 100 µm.
(2.85 MB TIF)

Figure S4 cxcr4a mRNA expression in odysseus fins (ody−/−) regenerating fins cxcr4a mRNA expression pattern was analyzed by in situ hybridization on control fin (wt) and on odysseus fins (ody−/−) after 1 or 3 dpa. No difference in expression of cxcr4 was observed between odysseus fins (ody−/−) and wild type fish.
(1.72 MB TIF)
