# Supplementary figures and images for: Fgf and Sdf-1 Pathways Interact during Zebrafish Fin Regeneration
Source: PLoS One. 2009 Jun 8;4(6):e5824. doi: 10.1371/journal.pone.0005824 (PMC2688747; doi:10.1371/journal.pone.0005824)

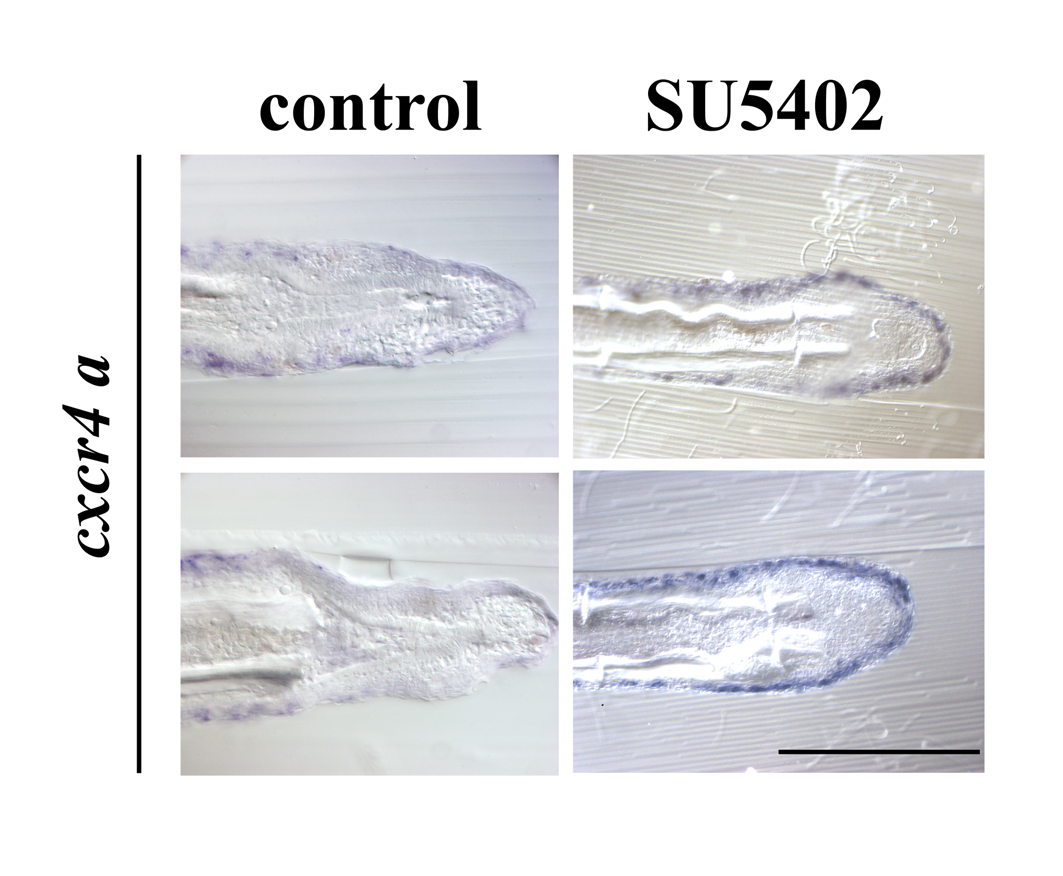

Supplement: Figure S1 — FgfR inhibition modifies, cxcr4a, expression in ongoing fin regenerates. Sections of 2 dpa caudal fins from fish treated with DMSO (control) or FGFR inhibitor (SU5402) after in situ hybridization for, cxcr4a. Scale bar, 100 µm. (2.83 MB TIF) [file pone.0005824.s001.tif]

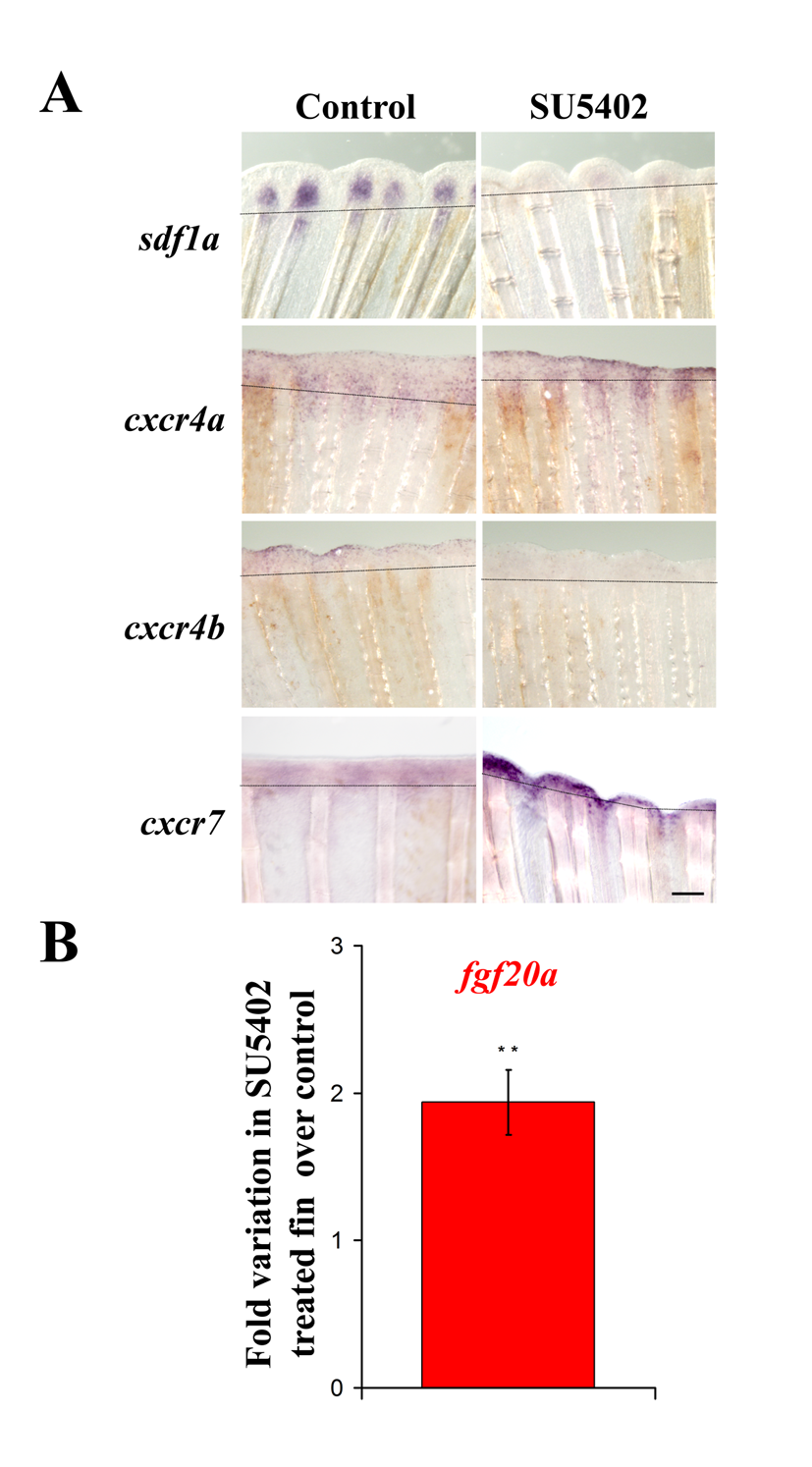

Supplement: Figure S2 — A. SU5402 treatment modifies sdf1, cxcr4a, cxcr4b and cxcr7 expression. mRNA expression was analyzed by in situ hybridization on 2 dpa caudal fins from fish treated with DMSO (control) or FGFR inhibitor (SU5402). Scale bar, 100 µm. B. fgf20a expression is enhanced in SU5402 treated fins : fgf20a expression was analyzed at 48 hpa by quantitative RT-PCR in SU540-treated fish and DMSO fins as control. fgf20a expression is increased 1,9-fold when the FGF pathway is blocked compared to control. Average values (±s.e.m.) from experiments performed in quadruplicate are presented (** p<0.01). (3.74 MB TIF) [file pone.0005824.s002.tif]

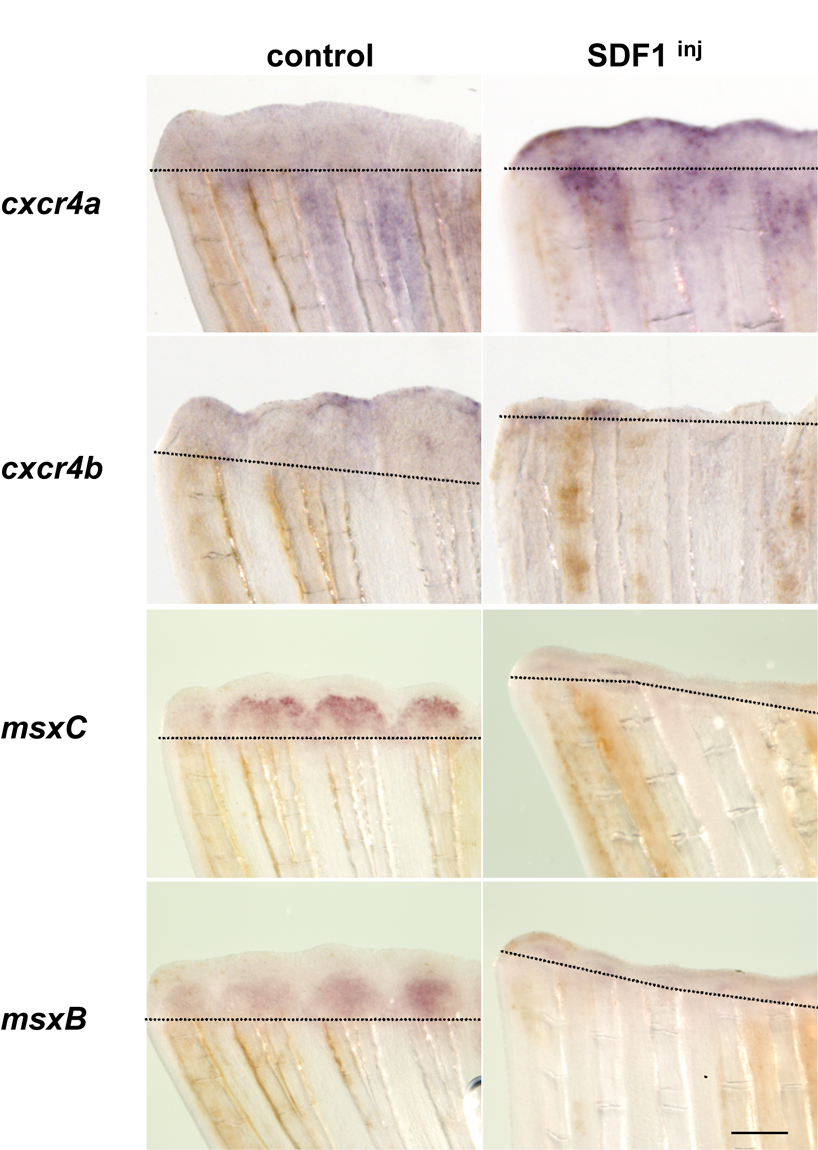

Supplement: Figure S3 — SDF1 overexpression inhibits Fgf downstream genes expression The SDF1 protein, or BSA as a control, were injected in the fin at the time of amputation. Fins were allowed to regenerate for 48 hours before being stained for Fgf20 downstream genes cxcr4a, cxcr4b, msxb and msxc expression. Dotted lines demarcate amputation plane. Scale bar, 100 µm. (2.85 MB TIF) [file pone.0005824.s003.tif]

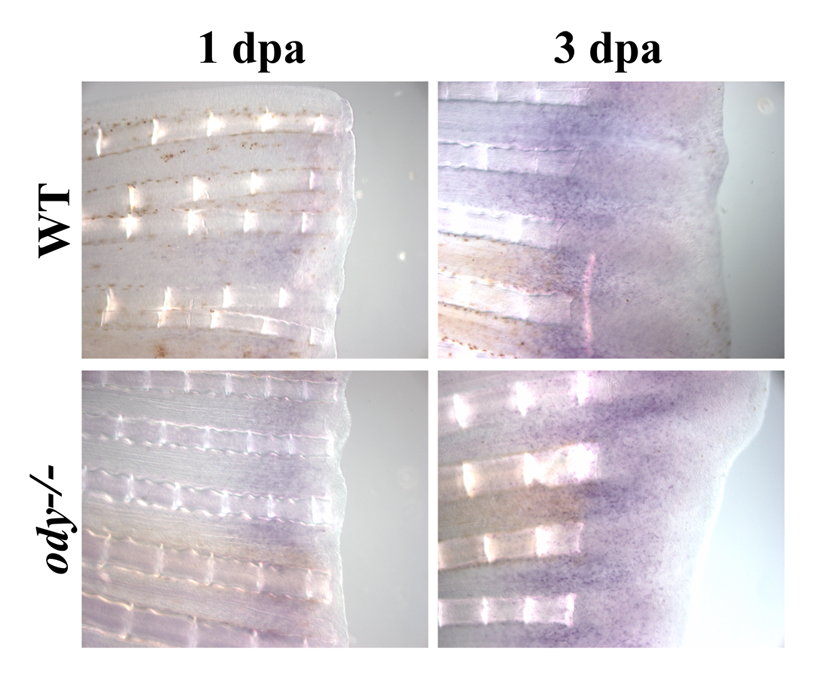

Supplement: Figure S4 — cxcr4a mRNA expression in odysseus fins (ody−/−) regenerating fins cxcr4a mRNA expression pattern was analyzed by in situ hybridization on control fin (wt) and on odysseus fins (ody−/−) after 1 or 3 dpa. No difference in expression of cxcr4 was observed between odysseus fins (ody−/−) and wild type fish. (1.72 MB TIF) [file pone.0005824.s004.tif]
